# Neurobiological correlates of Mild Behavioral Impairment: a systematic review and meta-analysis

**DOI:** 10.1186/s13195-025-01874-9

**Published:** 2025-10-21

**Authors:** Francesca Remelli, Maria Giorgia Barbieri, Elena Ferrighi, Federico Triolo, Giulia Grande, Davide Liborio Vetrano, Caterina Trevisan, Stefano Volpato

**Affiliations:** 1 Department of Medical Sciences, University of Ferrara, Via Savonarola 9, Ferrara, 44121 Italy; 2 Aging Research Center, Department of Neurobiology, Care Sciences and Society, Karolinska Institutet and Stockholm University, Tomtebodavägen 18 A, Solna, Stockholm, 17177 Sweden; 3 Stockholm Gerontology Research Center, Stockholm, Sweden; 4 Orthogeriatric Unit, University Hospital of Ferrara, Ferrara, Italy

**Keywords:** Mild behavioral impairment, Alzheimer’s disease, Dementia, Brain imaging, Positron emission tomography, Cerebrospinal fluid, Plasma, Biomarkers, Neurodegeneration, Neuroinflammation

## Abstract

**Background:**

Although individuals with Mild Behavioral Impairment (MBI) show an increased rate of developing dementia, it remains uncertain whether MBI should be considered a risk factor or an actual early sign of neurocognitive disease.

**Objectives:**

This systematic review and meta-analysis aimed to explore the association between MBI and neurobiological correlates of dementia.

**Methods:**

The study protocol followed PRISMA guidelines and was registered in PROSPERO (CRD42024589059). Five databases and gray literature were systematically searched from inception to January 31, 2025 to identify studies that explored the relationship between MBI and brain imaging findings or neurodegenerative and neuroinflammatory fluid biomarker levels. When studies employed comparable methodologies, a random-effects meta-analysis was performed to summarize the results; conversely, a qualitative synthesis was conducted. The Newcastle-Ottawa Quality Assessment Scale was used to assess the study quality.

**Results:**

Of the 834 records, 27 studies were included. Most studies were cross-sectional and examined the presence of structural or functional abnormalities through brain imaging in individuals with MBI. Six studies, 4 of which were longitudinal, focused on MBI and cerebrospinal fluid or plasma biomarkers of neurodegeneration and neuroinflammation. Due to the high methodological heterogeneity across studies, five random-effects meta-analyses were conducted, each including two studies. These analyses reported a positive, cross-sectional correlation between MBI burden and brain deposition of amyloid-beta (Aβ) or tau. Conversely, MBI was not significantly associated with either plasma phosphorylated-tau181 levels or Magnetic Resonance Imaging (MRI) brain atrophy markers. Nevertheless, based on the qualitative synthesis of the 27 included studies, MBI was frequently linked to Alzheimer’s disease (AD) abnormalities – both in biomarkers and brain imaging studies.

**Conclusions:**

Across studies, MBI appears to be linked to specific neurobiological markers of AD, including Aβ and tau brain deposition, as well as alterations in the mesolimbic pathway and neurodegenerative and neuroinflammatory fluid biomarker levels. Although emerging evidence supports MBI as a potential early clinical sign of AD, heterogeneity across studies precludes definitive conclusions regarding its precise role in the onset and progression of the disease.

**Supplementary Information:**

The online version contains supplementary material available at 10.1186/s13195-025-01874-9.

## Background

In light of the promising results of trials on disease-modifying therapies, detecting Alzheimer’s Disease (AD) in its early stages is crucial for maximizing the potential pharmacological benefits [1–5]. The scientific community recognizes Mild Cognitive Impairment (MCI) as the strongest clinical at-risk condition for incident dementia, despite its poor specificity [6, 7]. To support the diagnosis of early AD in individuals with suggestive cognitive deficits, structural and functional brain imaging, along with biological tests on cerebrospinal fluid (CFS), are often required [8]. Yet, the high cost of these techniques, the need for specialized medical personnel, and also the reduced access to advanced diagnostics, limit their universal availability [9, 10]. Thus, the introduction of simple, quick, and inexpensive disease markers may be extremely helpful in improving the early detection of AD in cognitively unimpaired individuals or those with MCI. On this regard, from a clinical perspective, adding the assessment of late-life neuropsychiatric symptoms to cognitive evaluation seems to be a valuable strategy for enhancing the prognostic performance of MCI and also detecting an at-risk condition for dementia in cognitively unimpaired individuals [11–16]. At this point, in accordance with the National Institute on Aging and Alzheimer’s Association (NIA-AA) research framework for AD [17], Mild Behavioral Impairment (MBI) was introduced to identify new and persistent late-onset neuropsychiatric symptoms associated with a high risk of all-cause dementia [18–20]. According to Alzheimer’s Association International Society to Advance Alzheimer’s Research and Treatment (ISTAART) diagnostic criteria [21], MBI was defined as a late-life neurobehavioral syndrome characterized by a sustained (greater than 6 months) change in usual behavior or personality, with at least minimal social or occupational impairment, without compromising daily individual autonomy [21]. During clinical assessment, MBI can be easily detected using an informant-rated scale called MBI-Checklist (MBI-C), which was designed to screen neuropsychiatric symptoms in cognitively healthy individuals with MCI [22–24]. A growing body of research on brain imaging and biomarkers suggests that MBI may be considered an actual anticipatory phase of dementia rather than merely an at-risk state for the disease; however, the available data remain inconsistent [25–29]. 

Our aim was to conduct a systematic review and meta-analysis to summarize the current evidence on the neurobiological correlates of MBI. Given the widely reported association between MBI and incident dementia, we hypothesize that the presence of new and persistent late-onset neuropsychiatric symptoms may be due to the accumulation of neurodegenerative processes.

## Methods

This work was conducted in accordance with the Preferred Reporting Items for Systematic Reviews and Meta-Analyses (PRISMA) [30] 2020 statement (checklist reported in Table S1) and recorded in PROSPERO with registration number CRD42024589059.

### Search strategy

The literature search was performed in 5 academic electronic databases (PubMed, Web Of Sciences, Embase, Cochrane Library, PsycINFO) and a gray literature database (EBSCO) from inception to January 31, 2025. The systematically reviewed studies explored the association between MBI and biological correlates of dementia. In particular, our research was driven by the following key question: *Do individuals with MBI exhibit any neurobiological features of dementia*,* and if so*,* do neurodegenerative or neuroinflammatory processes influence the onset of MBI and its progression to dementia over time?* The PECOS criteria identified to address these aims were: dementia-free individuals aged 50 years and over (Population), presence of MBI (Exposure), absence of MBI (Comparison), neurobiological correlates of dementia assessed by structural and functional brain imaging or CFS and blood levels of biomarkers (Outcome), cross-sectional or longitudinal studies (Study design). In the case of a cross-sectional study, the exposure and the outcome were considered interchangeable. Concerning the outcomes, biomarkers of both neurodegeneration (amyloid-beta 42, amyloid-beta 40, amyloid-beta 42/40 ratio, total-tau, phosphorylated-tau 181, phosphorylated-tau 217, Neurofilament Light chain) and neuroinflammation (e.g. Glial Fibrillary Acidic Protein, Soluble Triggering Receptor Expressed on Myeloid cells 2) were included. According to PECOS criteria, the search strategy included the following keywords: “Mild Behavioral Impairment” OR “Mild Behavioural Impairment”. The detailed search strategy for each database is reported in Table 2S.

### Inclusion criteria

All original peer-reviewed studies were retrieved. Research protocols, conference abstracts, editorials, and letters to the editors were excluded. No language restrictions were applied.

The inclusion criteria were the following: (1) original observational cross-sectional or longitudinal studies; (2) neurobiological assessment through brain imaging or CFS and blood biomarkers of neurodegeneration and neuroinflammation; (3) Diagnosis of MBI (or an individual MBI domain) according to ISTAART criteria; (4) focus on dementia-free individuals aged 50 years and older.

### Selection and data extraction

After deduplication, all articles were screened by two independent researchers (EF and MGB) based on titles and abstracts using Rayyan Software [31]. To evaluate eligibility, records that met the inclusion criteria were further assessed by the two independent researchers (EF and MGB) through the full-text review. In addition, the lists of references for each included article and relevant studies available on the topic of the review were retrieved. Any disputes were solved through a discussion with a third researcher (FR).

Data extraction was performed by three researchers (EF, MGB, FR) using a pre-designed form: first author, year of publication, country, study design, follow-up period (when applicable), study population, cohort (clinical vs. population-based), study name, age and sex distribution, prevalence of MCI, neuropsychiatric symptoms assessment tool, prevalence of MBI, cognitive performance in the study groups, neurobiological measures, and main findings. When the prevalence of MBI was not explicitly reported in the study, the mean scores of neuropsychiatric symptoms were extracted, when assessed using a standardized scale (e.g., the MBI-C or the Neuropsychiatric Inventory [NPI]).

### Quality assessment

Study quality assessment was performed using the Newcastle-Ottawa Scale (NOS) [32] specific for cross-sectional (Figure S1) and longitudinal studies (Figure S2).

### Data synthesis

A meta-analysis was performed for data with sufficiently homogenous types of methods and outcomes assessment, while a narrative synthesis was carried out for the other studies not included in the meta-analysis.

A random-effect meta-analysis was performed to estimate the standardized coefficients ($\:\beta\:$) and 95% confidence interval (95%CI) of the correlations between diagnosis of MBI (or MBI burden) and specific neurobiological measures (e.g., plasma phosphorylated-tau 181, hippocampal volume, thickness of entorhinal cortex, amyloid-beta and tau brain deposition). To harmonize the effect sizes across studies employing different MBI assessments, we first performed z-score transformation of the NPI-Q and MBI-C scores, and second a meta-analysis to estimate pooled standardized β coefficients. The between-studies heterogeneity was assessed through the Chi-squared test (considering a *p-*value < 0.10 as significant), and expressed by the *I*-squared statistic (*I*^2^), with a value >75% indicating high heterogeneity [33]. The meta-analysis was performed using the *meta* package of the R statistical program (version 2024.12.1) [34]. 

## Results

### Study selection

**Fig. 1:**
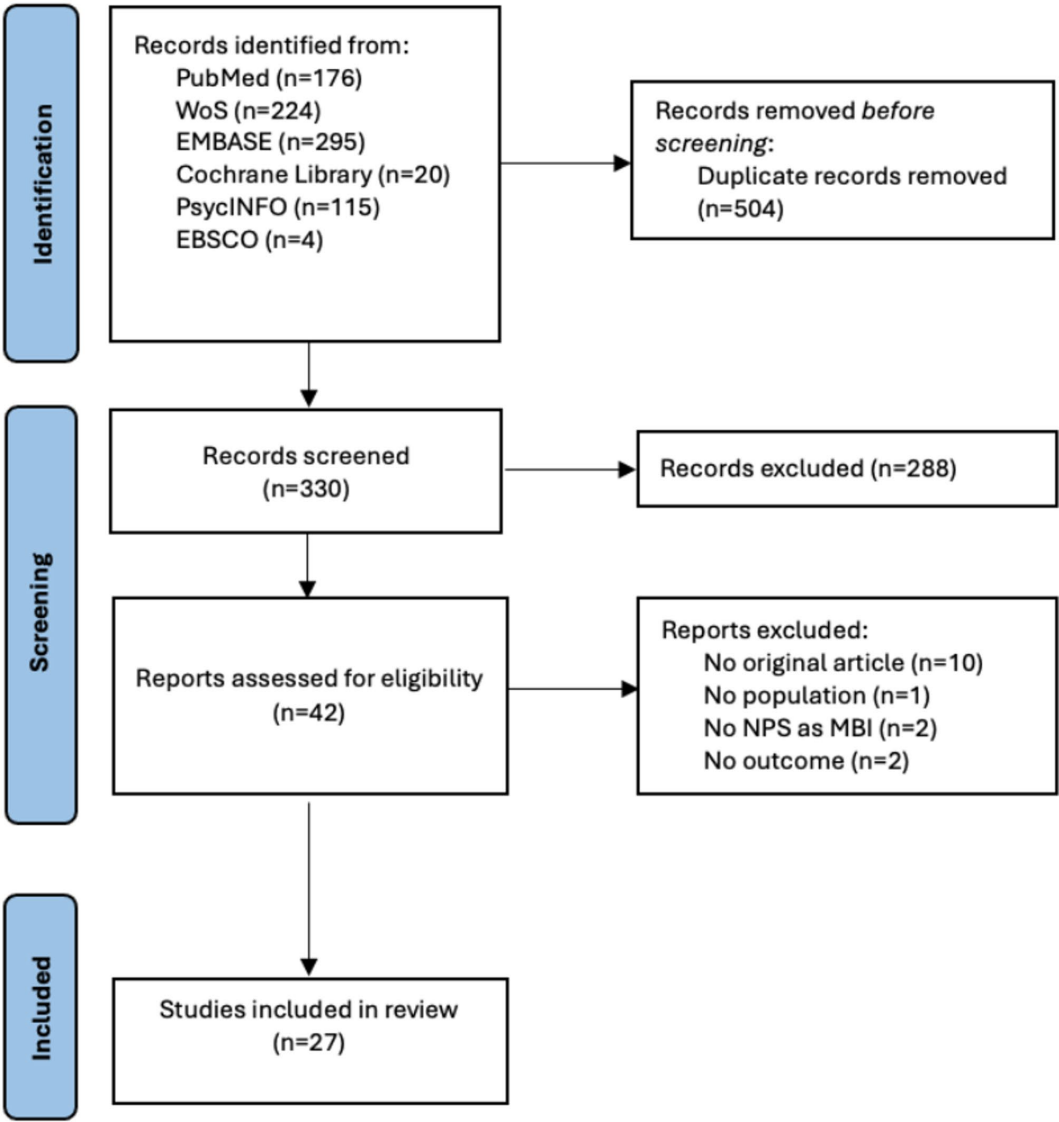
PRISMA flow diagram of study selection

A total of 834 records were initially identified by the search strategy and, after removing duplicates, 330 articles were screened based on the title and abstract. Of these, 42 were selected for full-text screening, and 27 met the eligibility criteria and were included in the review [25–29, 35–56]. The excluded studies were non-original articles (*n* = 10), conducted on individuals with dementia (*n* = 1), assessed neuropsychiatric symptoms without applying the MBI framework (*n* = 2), or explored different neurobiological outcomes related to dementia (*n* = 2). The study selection process is shown in Fig. 1.

### Characteristics of the selected studies

The main characteristics of the 27 included studies are shown in Table 1. Most had a cross-sectional design (*n* = 22); [25, 26, 28, 29, 36–42, 44, 45, 47–55] five were longitudinal [27, 35, 43, 46, 56]. Most of studies were conducted in Canada (*n* = 9) [25, 36, 41–44, 48, 49, 55], followed by six in Europe [26–29, 35, 56], four in China [45–47, 51], three in Japan [37, 39, 40], three in United States [38, 53, 54] and two in South Korea [50, 52]. Based on the NOS assessment, the overall quality was high (see Table S3). For the cross-sectional studies, NOS scores ranged from seven to ten points, with a median of eight; among these 22 studies, eight received the lowest observed score of seven, while two achieved the maximum score of ten. For the five longitudinal studies, the median NOS score was eight, with a range of seven to nine. Most studies were conducted on clinical cohorts (*n* = 24) [25–29, 35–39, 41–52, 54, 55], while three included population-based samples [40, 53, 56]. The sample sizes ranged from 34 to 1,445, with eleven studies reporting fewer than 100 participants; [25, 26, 40, 42, 45, 47–49, 51, 52, 55] the mean age was about 70 years. Most studies included individuals with MCI, with a prevalence ranging from 27.5% to 100.0%. Only two studies were conducted on cognitively unimpaired individuals [25, 45], while two other studies did not report this information but were conducted on dementia-free samples [47, 52]. Five studies were performed on cohorts with Parkinson’s Disease (PD) [39, 42, 48, 49, 55], one on individuals with mild stroke [51], and one on individuals with isolated REM sleep behavior disorder (iRBD) [52]. 

**Table 1:** Main characteristics of the 27 selected studies

Author, year (country)	Study design (follow-up period)	Study population (cohort), study name	Age (years), sex (F)	Prevalence of MCI	Prevalence of MBI (NPS assessment tool)	Cognitive performance in the study groups	Neurobiological measures	Main findings
**CFS and plasma biomarkers of neurodegeneration and neuroinflammation**								
Gonzalez-Bautista et al., 2024 (France) [56]	Longitudinal (1 years)	359 individuals *≥*70 years (population-based cohort), NOLAN Study	Mean age: 78.3 (*SD* 0.3), 58.2%	60.2%,	64.7% (NPI-Q)	NA	p-tau181, GFAP, homocysteine (plasma)	Abnormal perception was associated with a significantly faster increase in plasma p-tau181 ($\:\beta\:$ 0.638, 95%CI:0.087–1.188) and homocysteine ($\:\beta\:$ 0.570, 95%CI:0.107–1.033)
Ghahremani et al., 2023 (United Kingdom) [35]	Longitudinal (4 years)	571 individuals *≥* 55 years (clinical cohort), ADNI Study	Mean age: 72.1 (*SD* 7.1), 46.7%	64.8%	18.0% (NPI-Q)	Mean MMSE MBI: 28.4 (*SD* 1.4) No-MBI: 28.5 (*SD* 1.6)	p-tau181 (plasma)	MBI was associated with increasing levels of plasma p-tau181 over 4 years ($\:\beta\:$ 0.014, 95%CI:0.003–0.026)
Ismail et al., 2023 (France) [27]	Longitudinal (4 years)	510 individuals *≥* 55 years (clinical cohorts), ADNI and MEMENTO Studies	Mean age: 70.6 (*SD* 7.8), 44.3%	100.0%	ADNI 26.4% MEMENTO 45.6% (NPI-Q)	Mean MMSE ADNI MBI: 28 (*IQR* 27–29) No-MBI: 29 (*IQR* 27–29) MEMENTO MBI: 28 (*IQR* 27–29) No-MBI: 28 (*IQR* 27–29)	t-tau, p-tau, Aß-42/40 (CSF)	In ADNI, MBI was associated with lower CSFAß-42/40 ratio ($\:\beta\:$ -0.023, 95%CI:-0.032- -0.013), higher t-tau ($\:\beta\:$ 0.028, 95%CI:-0.012-0.034) and p-tau levels ($\:\beta\:$ 0.027, 95%CI:0.014–0.039) over 4 years. In MEMENTO, MBI was associated only with greater p-tau levels ($\:\beta\:$ 0.015, 95%CI:-0.003-0.027) over 4 years
Johansson et al., 2021 (Sweden) [26]	Cross-sectional	50 individuals [27] *≥* 40 years (clinical cohort), BF-2 Study	Mean age: 72.3 (*SD* 9.7), 50.0%	50.0% (including individuals with subjective cognitive decline)	Mean of MBI burden: 6.0 (IQR 12.0) (MBI-C)	N/A	tau brain levels (tau-PET) p-tau 181, Aß-42/40 (CFS)	The MBI-C total score was associated with the tau deposition in entorhinal cortex and hippocampus ($\:\beta\:$ 0.010, *SE* 0.003, *p* = 0.003) and the CSF p-tau 181 levels ($\:\beta\:$ 0.408, *SE* 0.142, *p* = 0.006)
Miao et al., 2022 (Canada) [41]	Cross- sectional	139 individuals *≥* 55 years (clinical cohort), ADNI study	Mean age: 72.4 (*SD* 7.6), 51.8%	61.9%	Mean of MBI burden: 0.54 (IQR 0–1) (NPI)	N/A	*Aß-42/40* (plasma)	Greater MBI burden and greater MBI affective dysregulation were associated with lower plasma *Aß-42/40* ($\:\beta\:$ -0.002, *SE* 0.001, *p* = 0.039 and $\:\beta\:$ -0.005, *SE* 0.002, *p* = 0.041, respectively).
Naude et al., 2020 (Canada) [43]	Longitudinal (2 years)	584 individuals *≥* 55 years (clinical cohort), ADNI Study	Mean age: 73.8 (*SD* 7.4), 46.1%	56.5%	34.7% (NPI-Q)	Prevalence of MCI MBI: 82.1% No-MBI: 44.2%	NfL (plasma)	Time*MBI status was the only significant interaction associated with change in NfL concentrations (*F*(1,574) = 4.59, *p* *=* 0.032) at 2 years
**Structural brain imaging**								
Gill et al., 2021 (Canada) [36]	Cross-sectional	203 individuals *≥* 55 years (clinical cohort), ADNI-GO/2 Study	Mean age: 73.3 (*SD* 6.8), 45.3%	46.8%	42.0% (NPI-Q)	Prevalence of MCI MBI: 45.3% No-MBI: 54.7%	Hippocampus, cingulate gyrus, fornix, fronto-occipital fasciculus, uncinate fasciculus (DTI) Hippocampus, anterior cingulate, amygdala, medial orbitofrontal cortex parahippocampal girus (MRI)	Impulse dyscontrol was associated with white matter abnormalities of fornix, superior fronto-occipital fasciculus, cingulum and uncinate fasciculus, and with a smaller cortical thickness in the parahippocampal gyrus ($\:\beta\:$ -0.1, *SE* 0.04, *p* = 0.008)
Guan et al., 2024 (United States) [54]	Cross-sectional	1,273 individuals *≥* 50 years (clinical cohort), NACC Data Platform	Mean age: 69.8 (*SD* 9.8), 60.3%	27.5%	11.5% (NPI-Q)	Prevalence of MCI MBI: 29.1% No-MBI: 22.0%	Hippocampus, entorhinal cortex (MRI)	MBI was associated with a lower hippocampus volume both in MCI group and cognitively intact group ($\:\beta\:$ -0.52,95%CI:-0.88- -0.17, and $\:\beta\:$ -0.38,95%CI:-0.67- -0.09, respectively), while with a smaller entorhinal cortex only in MCI group ($\:\beta\:$ -0.37,95%CI:-0.63- -0.10)
Imai et al., 2023 (Japan) [37]	Cross-sectional	122 individuals *≥* 50 years (clinical cohort)	Mean age: 76.2 (*SD* 7.4), 67.2%	73.7%	36.8% (ISTAART criteria by retrospective review of medical records)	Mean MMSE MBI: 26.0 (*SD* 2.6) No-MBI: 26.2 (*SD* 2.5)	Supramarginal gyri, bilateral parahippocampal gyri, angular gyri, superior parietal lobule, and middle frontal gyri (MRI)	MBI was associated with a smaller cortical thickness in the right supramarginal gyrus (MBI 2.38 ± 0.16 vs. non-MBI 2.48 ± 0.14, *p* = 0.002, η²=0.02)
Liu et al., 2024 (China) [51]	Cross-sectional	72 individuals *≥* 50 years with mild stroke (clinical cohort)	Mean age: 68.9 (*SD* 13.0), 29.2%	NA	36.1% (MBI-C)	NA	Precuneus cortical thickness (MRI)	A thinner precuneus cortex was associated with a higher risk of MBI (OR 0.02, 95%CI:0.00-0.39)
Lussier et al., 2020 (Canada) [25]	Cross-sectional	96 individuals *≥* 55 years (clinical cohort), TRIAD Study	Mean age: 71.5 (*SD* 6.0), 60.4%	0.0%	Mean of MBI burden: 1.9 (*SD* 4.4) (MBI-C)	N/A	A*β* brain levels (Aβ-PET) Gray matter volume (MRI)	Higher MBI-C scores predicted higher global 𝛽-amyloid PET uptake (*R* = 0.27, *p* < 0.001), especially in the left frontal cortex, left posterior cingulate cortex, left caudate nucleus, and left thalamus. No significant correlations were found between MBI-C score and gray matter volume
Matuskova et al., 2021 (Czech Republic) [28]	Cross-sectional	116 individuals *≥* 55 years with subjective cognitive concerns (clinical cohort), CBAS Study	Mean age: 69.6 (*SD* 8.2), 49.0%	68.1%	Mean of MBI burden: 4.5 (*SD* 5.4) (MBI-C)	N/A	Hippocampus, entorhinal cortex (MRI)	Entorhinal cortex was associated with MBI-C total score (*r*_*S*_= -0.284, *p* = 0.002)
Miao et al., 2021 (France) [29]	Cross-sectional	768 individuals *≥* 60 years (clinical cohort), MEMENTO Study	Mean age: 72.8 (*SD* 8.0), 57.0%	100.0%	29.8% (NPI)	Mean MMSE MBI: 27.1 (*SD* 2.3) No-MBI: 28.0 (*SD* 1.8)	White matter hyperintensities (MRI)	MBI + status was associated with a higher WMH volume than MBI- status ($\:\beta\:$ 0.094, 95%CI:0.002–0.167)
Monchi et al., 2024 (Canada) [42]	Cross-sectional	91 individuals with PD (clinical cohort)	Mean age: 70.2 (*SD* 7.0), 60.1%	41.8%	24.2% (MBI-C)	Prevalence of MCI MBI: 40.9% No-MBI: 42.0%	Connections between subcortical and frontal regions, as superior, middle, inferior and orbitofrontal gyri, as well as connections between subcortical regions, as hippocampus, amygdala, nucleus accumbens, caudate nucleus, and putamen (DTI)	Compared to those with PD-non-MBI, individuals with PD-MBI the ipsilateral connection between the left amygdala and the putamen was disrupted, as shown by decreased both fixel-based apparent fiber density (*η*^2^ = 0.05, *p* = 0.048) and fractional anisotropy (*η*^2^ = 0.12, *p* = 0.014), and increased tissue radial diffusivity (*η*^2^ = 0.09, *p* = 0.004)
Shu et al., 2021 (China) [45]	Cross-sectional	34 individuals *≥* 50 years (clinical cohort)	Mean age: 67.0 (*SD* 6.9), 50.0%	0.0%	47.1% (MBI-C)	Mean MMSE MBI: 28.2 (*SD* 1.2) No-MBI: 28.8 (*SD* 0.8)	Brainstem, temporal transverse gyri, thalamus, superior and inferior temporal gyri, occipital pole, precentral gyri, middle temporal gyri, middle frontal gyri, white matter hyperintensities (MRI)	MBI was associated with decreased gray matter volume in the left brainstem (t= 4.56, *p* < 0.001), bilateral temporal cortex (t= 4.54, *p* < 0.001), right occipital pole (t= 4.14, *p* < 0.001), right thalamus (t= 4.23, *p* < 0.001), and left frontal cortex (t= 3.77, *p* < 0.001)
Yang et al., 2022 (China) [47]	Cross-sectional	60 individuals *≥* 50 years (clinical cohort)	Mean age: 69.5 (*SD* 8.5), 51.7%	NA	Mean of MBI burden: 5.3 (*SD* 4.9) (MBI-C)	N/A	Gray matter volume and white matter hyperintensities (MRI)	MBI-C score was negatively correlated with left anterior insula (R^2^ = 0.778, p​<​0.05), left thalamus gray matter volume (R^2^ =​0.615, p<​0.05,) and right posterior cingulate cortex thickness (R^2^​=0.779, p​<​0.05)
Yoon et al., 2019 (Canada) [48]	Cross-sectional	60 individuals *≥* 30 year with PD (clinical cohort)	Mean age: 70.6 (*SD* 6.3), 30.0%	53.3%	30.0% (MBI-C)	Mean MoCA MBI: 23.3 (*SD* 4.3) No-MBI: 27.1 (*SD* 2.4)	Cortical thickness, bilateral caudate nucleus, putamen, pallidum, nucleus accumbens, hippocampus, amygdala, thalamus (MRI)	The group with PD-MBI showed thinning (*p* < 0.001) and decreased volume (*p* = 0.043) in the right middle temporal cortex compared to the PD-noMBI group, and thinning in the left parahippocampal cortex (*p* = 0.004), decreased volume in right precuneus (*p* = 0.040), frontal cortex (*p* = 0.002) and left parietal lobule (*p* < 0.001) compared to healthy controls
Yoon et al., 2023 (South Korea) [50]	Cross- sectional	1,184 individuals *≥* 60 years (clinical cohort)	Mean age: 73.5 (*SD* 5.7), 61.7%	100.0%	52.6% (NPI-Q)	NA (MCI study population)	Cortical thickness, bilateral caudate nucleus, putamen, pallidum, nucleus accumbens, hippocampus, amygdala, thalamus (MRI)	The multiple co-occuring MBI domains in individuals with amnestic MCI is associated with higher cortical thinning bilaterally in the inferior parietal, lateral occipital, lateral superior temporal, and frontopolar regions (*p* < 0.01)
Young et al., 2024 (United States) [53]	Cross- sectional	1,445 individuals *≥* 45 years (population based cohort), ARIC Study	Mean age: 76.5 (*SD* 5.2), 59.0%	40.0%	26.0% (NPI-Q)	Prevalence of MCI MBI: 47.0% No-MBI: 37.0%	Entorhinal cortex, hippocampus, left postcentral gyrus, left superior frontal gyrus, left middle frontal gyrus (MRI)	MBI was associated with lower gray matter volume, predominantly in posterior cerebellar and bilateral temporal lobe structures (*p* < 0.001), including the hippocampus
**Functional brain imaging**								
Iordan et al., 2024 (United States) [38]	Cross-sectional	128 individuals *≥* 55 years (clinical cohort), STIM Study	Mean age: 72.4 (*SD* 6.7), 48.4%	100.0%	Mean of MBI burden: 2.13 (*SD* 2.79) (NPI-Q)	N/A	A*β* brain levels (Aβ-PET) tau brain levels (tau-PET) Canonical salience, default-mode, and frontoparietal control networks (resting-state fMRI)	Lower overall segregation of the salience network was associated with more severe MBI symptoms ($\:\beta\:$ -0.441, *SE* 0.178, *p* = 0.013)
Lang et al., 2020 (Canada) [55]	Cross-sectional	74 individuals with PD (clinical cohort)	Mean age: 70.9 (*SD* 6.0), 33.8%	43.2%	28.4% (MBI-C)	Mean MoCA MBI: 28.8 (*SD* 4.3) No-MBI: 26.0 (*SD* 3.5)	Canonical salience, default-mode, frontoparietal control and striatal networks (resting-state fMRI)	PD-MBI is associated with altered corticostriatal connectivity, particularly between the head of the caudate (*p* = 0.0085) and cortical (*p* = 0.0132) regions
Yoon et al., 2021 (Canada) [49]	Cross-sectional	59 individuals with PD (clinical cohort)	Mean age: 70.2 (*SD* 6.4), 37.3%	35.6%	35.6% (MBI-C)	Mean MoCA MBI: 23.5 (*SD* 4.4) No-MBI: 26.6 (*SD* 2.4)	Activation of brain cortex and subcortical regions (fMRI during performance)	PD-MBI group revealed less activation in the prefrontal and posterior parietal cortex, and reduced deactivation in the medial temporal area than PD-noMBI group
Matsuoka et al., 2021 (Japan) [40]	Cross-sectional	43 individuals *≥*50 years (population-based cohort)	Mean age: 76.9 (*SD* 5.7), 53.4%	30.2%	Mean of MBI burden: 2.7 (*SD* 4.2) (MBI-C)	N/A	Canonical salience, default-mode, and frontoparietal control networks (resting-state fMRI)	A negative correlation was observed between the MBI-C total score and frontoparietal control network of the left posterior parietal cortex with the right middle frontal gyrus (t= -4.39, FDR-corrected *p* *=* 0.015). Similar findings were found in relation to MBI-C affective dysregulation score (t=-4.14, FDR-corrected *p*= 0.032)
Matsuoka et al., 2023 (Japan) [39]	Cross-sectional	103 individuals *≥* 50 years with AD or Parkinson’s spectrum disorders (clinical cohort), PADNI Study	Mean age: 69.3 (*SD* 15.8), 46.6%	31.1%	27.2% (NPI-Q)	NA	A*β* brain levels (Aβ-PET) Nigrostriatal dopaminergic function (DAT-SPECT)	In individuals with amyloid-positive and abnormal DAT-SPECT, the MBI abnormal perception score was higher compared to those with amyloid-negative and normal DAT-SPECT (Bonferroni-corrected *p* = 0.012)
Naude et al., 2024 (Canada) [44]	Cross- sectional	442 individuals *≥* 55 years (clinical cohort), ADNI Study	Mean age: 74.8 (*SD* 7.6), 50.9%	35.7%	12.2% (NPI-Q)	Prevalence of MCI MBI: 42.6% No-MBI: 29.8%	A*β* brain levels (Aβ-PET) tau brain levels (tau-PET)	Among A*β* positive individuals, MBI was associated with tau uptake in Braak I ($\:\beta\:$ 0.45, *SD* 0.15, *p* *<* 0.01) and Braak III ($\:\beta\:$ 0.24, *SD* 0.07, *p* < 0.01) regions
Sun et al., 2021 (China) [46]	Longitudinal (6 years)	1,129 individuals *≥* 55 years (clinical cohort), ADNI Study	Mean age: 72.0 (*SD* 7.0), 49.9%	48.1%	68.2% (NPI-Q)	N/A	A*β* brain levels (Aβ-PET) tau brain levels (tau-PET) Cerebral hypometabolism (FDG-PET)	Cross-sectionally, individuals with higher MBI total scores had greater β-amyloid (*β* 0.018, *p* = 0.006) burden and lower FDG PET (*β* − 0.020, *p* < 0.001); no significant associations with tau PET were found. Longitudinally, higher MBI total scores predicted higher β-amyloid deposition (*β* 0.003, *p* < 0.001)
Yoon et al., 2025 (South Korea) [52]	Cross- sectional	36 individuals *≥* 50 years with isolated REM sleep behavior disorder (clinical cohort)	Mean age: 71.5 (*SD* 6.1), 50.0%	NA	18.1% (MBI-C)	NA	A*β* brain levels (Aβ-PET)	Amyloid deposition in the bilateral anterior cingulate (left: t= 8.31, *p* < 0.001, right: t= 4.32, *p* < 0.001) and prefrontal (left: t= 7.06, *p* < 0.001, right: t= 5.10, *p* < 0.001), left orbitofrontal cortex (t= 5.98, *p* < 0.001), caudate nucleus (t= 6.31, *p* < 0.001), and putamen prefrontal (left: t= 5.59, *p* < 0.001, right: t= 5.27, *p* < 0.001) showed significant higher values in the MBI group

MBI = Mild Behavioral Impairment; MBI-C = Mild Behavioral Impairment Checklist; MCI = Mild Cognitive Impairment; AD = Alzheimer’s Disease; PD = Parkinson’s Disease; HC = Healthy Cognitive; t-tau = total-tau; Aβ42/40 = amyloid-beta 42/40 ratio; p-tau181 = phosphorylated-tau181; NfL = neurofilament light chain; GFAP = glial fibrillary acidic protein; CFS = Cerebrospinal fluid; WMH = White Matter Hyperintensities; GMV = Gray Matter Volume; FPCN = Frontoparietal Control Network; FC = Functional Connectivity; AFD = Apparent Fiber Density; RTD = Tissue Radial Diffusivity; FAT = Fractional Anisotropy; PFC = Prefrontal Cortex; MRI = Magnetic Resonance Imaging; dMRI = diffusion magnetic resonance imaging; fMRI = functional magnetic resonance imaging; DTI = Diffusion Tensor Imaging; FDG-PET = 18 F-fluorodeoxyglucose Positron Emission Tomography; tau-PET = tau Positron Emission Tomography; Aβ-PET = amyloid Positron Emission Tomography; DAT-SPECT = Dopamine Transporter Single Photon Emission Computed Tomography; NOLAN = Naproxen or Loratadine and Neulasta; ADNI = Alzheimer’s Disease Neuroimaging Initiative; NACC = National Alzheimer’s Coordinating Center; STIM = Stimulation to Improve Memory; BF-2 = Swedish BioFINDER − 2; TRIAD = Translational Biomarkers of Aging and Dementia; PADNI = Parkinson’s and Alzheimer’s disease dimensional neuroimaging initiative; CBAS = Czech Brain Aging study; ARIC = Atherosclerosis Risk in Communities; MMSE = Mini Mental State Examination; MoCA = Montreal Cognitive Assessment; SD = Standard Deviation; IQR = Interquartile range; SE = Standard Error; OR = Odds Ratio; 95%CI = 95% Confidence Interval; FDR = False Discovery Rate; NA = not available (missing data); N/A = not applicable

### Mild behavioral impairment

To assess neuropsychiatric symptoms, fourteen studies used NPI in its complete [29, 41, 44] or brief (i.e. NPI-Q) version [27, 35, 36, 38, 39, 43, 44, 46, 50, 53, 54, 56], twelve used MBI-C (*n* = 12) [25, 26, 28, 40, 42, 45, 47–49, 51, 52, 55], and one a retrospective detection through the review of medical records [37]. Except for the latter, the assessment scales for neuropsychiatric symptoms were completed by the informant. Based on the detected neuropsychiatric symptoms [22, 57], MBI was diagnosed according to the ISTAART criteria [21] and its prevalence across studies ranged from 18.1% to 68.2%. In seven studies [25, 26, 28, 38, 40, 41, 47], MBI was explored through the MBI burden, which was obtained by the number and severity of neuropsychiatric symptoms at the neuropsychiatric evaluation. When MBI-C was used [25, 26, 28, 40, 47], the mean of MBI burden ranged from 1.9 to 6.0, while 2.13 (*SD* 2.79) was reported through NPI-Q [38] and 0.54 by NPI [41]. 

### MBI and CFS and plasma biomarkers of neurodegeneration and neuroinflammation

Six studies explored the association between MBI and biomarkers measured in CSF [26, 27] or plasma [35, 41, 43, 56]. Most of them had a longitudinal design (*n* = 4) [27, 35, 43, 56], while two were cross-sectional [26, 41]. 

Concerning CFS biomarkers, in a sample of 510 MCI participants, MBI was cross-sectionally associated with lower levels of amyloid-beta 42 (Aß-42) and higher phosphorylated-tau (p-tau); additionally, participants with MBI showed a faster increase of p-tau levels over 4 years, without any significant longitudinal changes in Aß-42 [27]. The cross-sectional association with CFS phosphorylated-tau181 (p-tau181) was also reported by Johansson et al., both among cognitively unimpaired individuals (50.0%) and among those with MCI (50.5%), where a higher MBI burden was linked to elevated p-tau181 levels [26]. 

Regarding plasma biomarkers, two studies investigated longitudinal changes in p-tau181 levels [35, 56]. In the Alzheimer’s Disease Neuroimaging Initiative (ADNI) study, which included a sample of individuals with MCI [35], MBI was associated with a steeper increase in plasma p-tau181 over 4 years. This result was partially confirmed by Gonzalez-Bautista et al., [56] who reported a greater 1-year increase in plasma p-tau181 only in individuals with MBI abnormal perception. These individuals also showed a faster increase in homocysteine over time without any significant changes in Glial Fibrillary Acidic Protein (GFAP) levels. On plasma Aß biomarkers, both a greater overall MBI burden and MBI affective dysregulation were cross-sectionally associated with lower amyloid-beta 42/40 (Aß-42/40) ratio levels [41]. Lastly, concerning plasma Neurofilament Light Chain (NfL), in a clinical cohort of 584 individuals – with a 56.6% prevalence of MCI – MBI was associated with a steeper increase in NfL levels over a two-year period [43]. 

**Fig. 2:**
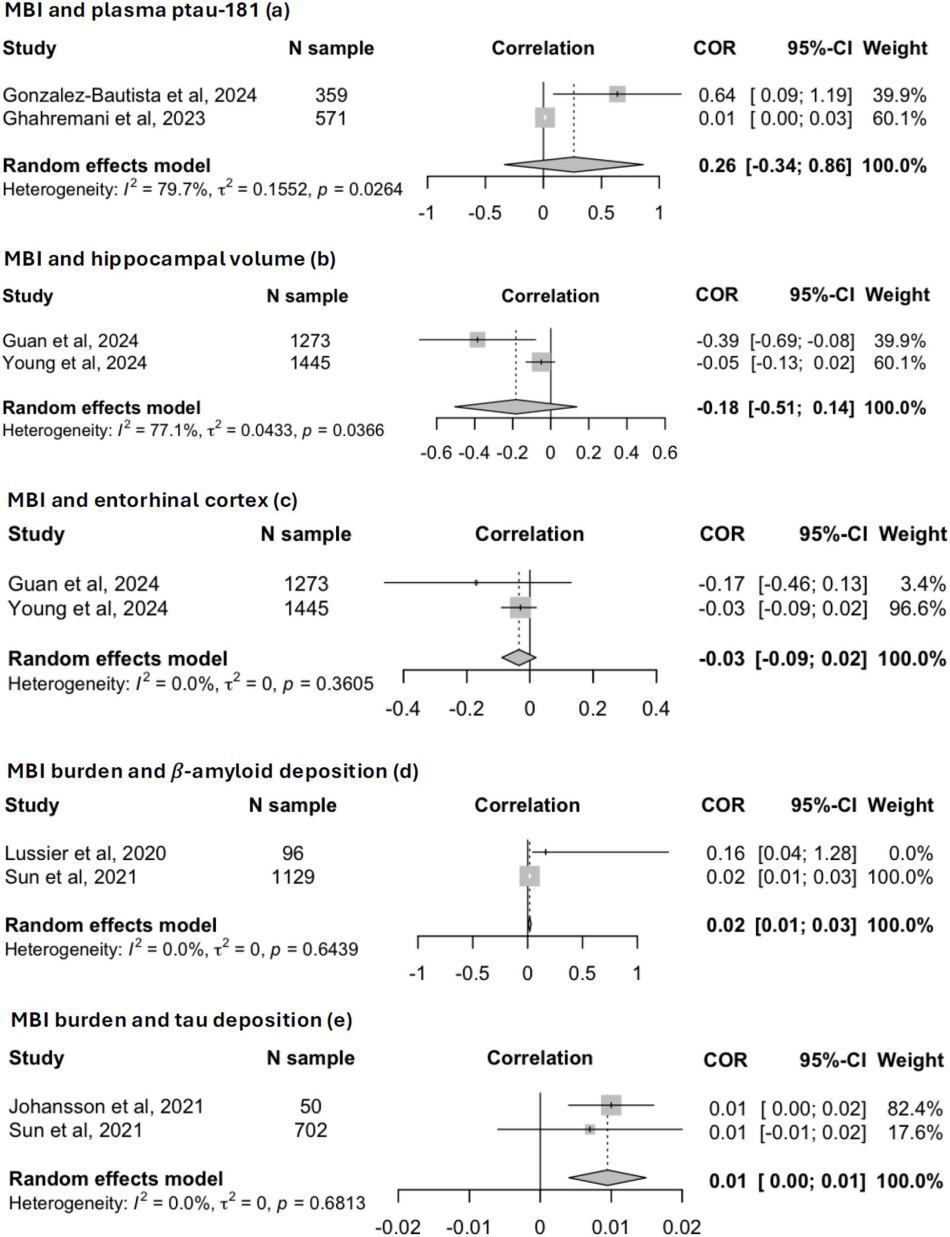
Forest plots showing pooled changes in neurobiological findings according to MBI status

Concerning the two longitudinal studies on MBI and plasma p-tau181 [35, 56], the random effects model, which included 930 participants, did not highlight a significant association between the presence of baseline MBI and changes in p-tau181 levels over time (standardized $\:\beta\:$ = 0.26, 95%CI: -0.34 to 0.86; *I*^2^ = 79.7%, *p* = 0.026); see Fig. 2 (Panel a).

### MBI and structural brain imaging

Thirteen studies measured brain volumes by Magnetic Resonance Imaging (MRI) in individuals with MBI [25, 26, 28, 29, 36, 37, 45, 47, 48, 50, 51, 53, 54]. All these studies were cross-sectional, and only one was conducted on a population-based cohort [53]. In most studies that used MRI to assess gray matter volume, MBI was consistently associated with reduced volumes and cortical thickness of specific brain areas related to AD. Particularly, six studies showed the presence of a smaller hippocampus and thinner entorhinal cortex in individuals with MBI than those without MBI [28, 36, 48, 50, 53, 54], especially when MCI co-occurred [54]. In addition, smaller cortical thickness of the bilateral temporal gyri [45, 48, 50, 53], frontopolar regions [45, 48, 50], precuneus [48, 51] and right supramarginal girus [37, 50] were described in participants with MBI. Two studies also reported an association between MBI and reduced thalamic volumes [45, 47], while no significant differences were observed in the basal ganglia [36, 50]. Lastly, in a large population-based study by Young et al. [53], the diagnosis of MBI was associated with lower global gray matter volume. However, this finding was not consistent with research on a clinical sample (*N* = 96) in which MBI burden was used as exposure [25]. Among MRI studies, three investigated the relationship between MBI and white matter hyperintensities with discordant results [29, 45, 47]. The higher matter hyperintensities volume in those with MBI, described by Miao et al. in a cohort of 768 individuals [29], was not found by Shu et al. in their study on cognitively unimpaired sample (*N* = 34) [45]. Additionally, when exploring MBI as a neuropsychiatric burden, such an association was not further confirmed [47]. 

Across the selected studies, two assessed the integrity of white matter tracts using Diffusor Tension Imaging (DTI) [36, 42]. In the clinical cohort studied by Gill et al., the presence of MBI impulse dyscontrol was associated with white matter abnormalities of the fornix, fronto-occipital fasciculus, cingulum, and uncinate fasciculus [36]. Limbic system injuries in MBI were partially confirmed in another study on a PD sample, which reported an impaired connection between the left amygdala and ipsilateral putamen [42]. 

The random-effects meta-analyses conducted on the studies by Guan et al. [54] and Gill et al., [36] which included 2,718 participants, found that MBI was not significantly associated with either lower hippocampal volume (standardized coefficient = -0.18, 95%CI: -0.51 to 0.14; *I*^2^ = 77.1%, *p* = 0.037) or a reduced thickness of entorhinal cortex (standardized $\:\beta\:$ = -0.03, 95%CI: -0.09 to 0.02; *I*^2^ = 0.0%, *p* = 0.361); see Fig. 2 (Panel b & c).

### MBI and functional brain imaging

Ten studies evaluated the presence of functional brain abnormalities in individuals with MBI through functional MRI (fMRI) [38, 40, 49, 55], amyloid Positron Emission Tomography (Aβ-PET) [25, 38, 39, 44, 46, 49, 52], tau Positron Emission Tomography (tau-PET) [26, 38, 44, 46], 18 F-fluorodeoxyglucose Positron Emission Tomography (FDG-PET) [46] and Dopamine Transporter Single Photon Emission Computed Tomography (DAT-SPECT) [39]. 

To investigate neural network integrity in MBI, three studies employed resting-state fMRI [38, 40, 55], whereas one study utilized fMRI during task performance [49]. Resting-state data showed that a higher MBI burden was related to lower segregation of the salience [38] and frontoparietal control networks [40]. In PD individuals, the presence of MBI was associated with greater impairment in corticostriatal connectivity [55]. Furthermore, during a set-shifting task, the MBI group exhibited reduced activation in the prefrontal and posterior parietal cortex, along with attenuated deactivation of the medial temporal region [49]. 

Six of the included studies explored the Aβ brain deposition in individuals with MBI [25, 38, 39, 44, 46, 52]. A higher MBI burden was cross-sectionally linked to a greater Aβ brain accumulation, particularly in the cingulate and frontal cortex and in the caudate nucleus; [25, 46] such an association was also found assessing the Aβ load over 6 years [46]. In contrast, a study on 128 MCI participants found no significant differences in mean baseline MBI burden between participants with and without Aβ PET positivity [38]. However, in individuals with iRBD, the presence of MBI was associated with increased Aβ brain deposition [52]. Additionally, Aβ PET positive individuals with concomitant dopaminergic deficits on DAT-SPECT exhibited a greater burden of MBI symptoms related to abnormal perception [39]. 

As reported in Fig. 2 (Panel d), the random-effects meta-analysis conducted on 1,225 participants [25, 46] showed a significant positive correlation between MBI burden and brain Aβ deposition (standardized $\:\beta\:$ = 0.02, 95%CI: 0.01 to 0.02) with low between-study heterogeneity (*I*^2^ = 0.0%, *p* = 0.361).

Four studies investigated tau pathology using PET imaging in individuals with MBI [26, 38, 44, 46]. Cross-sectional analyses revealed that higher MBI burden was associated with increased tau deposition in the entorhinal cortex and hippocampus [26, 44], particularly among individuals who were Aβ positive [44]. This association was partially replicated by Sun et al., although the results did not reach statistical significance [46]. Furthermore, the only available longitudinal data did not confirm a predictive link between baseline MBI burden and tau accumulation over six years [46]. The study by Sun et al. represents the only FDG-PET investigation conducted to date. In the clinical cohort of 1,129 individuals, the authors reported that higher MBI was cross-sectionally–though not longitudinally–associated with cerebral hypometabolism [46]. 

As shown in Fig. 2 (Panel e), a greater MBI burden was also associated with higher tau brain levels in the random-effects meta-analysis on 752 participants [26, 46] (standardized $\:\beta\:$ = 0.01, 95%CI: 0.00 to 0.01); the between-study heterogeneity was low (*I*^2^ = 0.0%, *p* = 0.681).

## Discussion

This systematic review of 27 studies explored the neurobiological correlates of MBI. Despite the limited the number of studies included in the quantitative synthesis, pooled analyses of comparable data revealed that a higher MBI burden was associated with increased Aβ and tau brain deposition. In contrast, no significant associations were observed between MBI and both plasma p-tau181 levels and brain volumes. Nevertheless, when all 27 studies examined in the qualitative synthesis were considered, MBI was found to be associated – both cross-sectionally and longitudinally – with a range of neurobiological markers characteristic of dementia, particularly AD.

### MBI and CFS and plasma biomarkers of neurodegeneration and neuroinflammation

Across the included studies, individuals with MBI had more likely abnormal CFS and plasma levels of biomarkers of neurodegeneration. Notably, although not confirmed by the meta-analysis of cross-sectional data, two studies [27, 35] reported that individuals with MBI exhibited a faster increase in p-tau levels over time both in CFS and in plasma, supporting an association between the presence of MBI and subsequent AD-related neurodegenerative processes. Nevertheless, no longitudinal evidence was found on the plasma Aß-42/40 ratio, which was only cross-sectionally associated with a greater MBI burden [27, 41]. As widely reported in the literature, higher CFS and plasma p-tau181 levels – specific to AD [60] – are linked to both tau and Aβ brain deposition, along with accelerated brain atrophy over time [61]. Thus, a steeper increase in p-tau181 over time in individuals with MBI supports underlying AD processes that may explain the faster progression to dementia, particularly when MCI co-occurrs [11, 16] Otherwise, following this hypothesis, the non-significant association between MBI and longitudinal changes in the plasma Aβ-42/40 ratio [27, 41] might be attributed to potential interfering factors affecting amyloid metabolism, such as cerebral amyloid angiopathy, elevated neuroinflammation, and nicotine exposure [62–64]. 

Based on our results, higher plasma NfL levels over 2 years were reported in individuals with MBI by Naude et al. [43] Although not specific to AD, NfL is a biomarker of neurodegeneration released in response to axonal injury associated with a faster progression of dementia [61, 65]. Thus, our finding supports the hypothesis that MBI may identify a clinical phenotype characterized by accelerated neurodegeneration and disease progression, also capturing dynamic changes in plasma NfL over time. Concerning biomarkers of neuroinflammation – which remains poorly investigated in the selected studies – the only available research found no association between MBI and longitudinal changes in plasma GFAP [56]. Nevertheless, the authors reported a faster 1-year increase in homocysteinemia in individuals with MBI, potentially linking this phenotype to the vascular component of dementia [56]. 

### MBI and structural brain imaging

Although data from meta-analyses did not yield significant results [53, 54], based on the qualitative synthesis, most selected research reported that individuals with MBI frequently exhibited reduced gray matter volumes with specific cortical patterns related to AD. The presence of temporal atrophy in MBI, particularly lower hippocampal volumes and entorhinal cortical thickness, was widely described across the selected studies [28, 36, 48, 50, 53, 54]. Moreover, reduced thickness of precuneus and right supramarginal girus, cortical regions involved in early AD [66], was cross-sectionally associated with MBI in four studies [37, 48, 50, 51]. Lastly, exploring white matter integrity using DTI, MBI appears to be associated with limbic system injuries, particularly in individuals with MBI impulse dyscontrol [36, 42]. However, due to the cross-sectional design of the included studies, no definitive conclusions can be drawn regarding the direction of the association between the presence of MBI and early neuropathological changes characteristic of AD. Among the deep gray matter structures, lower volumes of the thalamus in MBI were described in two studies, and this may be linked to AD or other types of dementia (i.e., Vascular Dementia - VaD) [67]. Nevertheless, three studies investigating leukoaraiosis on brain MRI yielded conflicting results regarding the association between MBI and matter hyperintensities, which was not further confirmed [29, 45, 47]. Moreover, across studies [45, 48, 50], consistent findings of reduced frontal cortical thickness in individuals with MBI support the hypothesis that the neurobiological underpinnings of MBI – particularly when characterized by frontal atrophy – could also be linked to Frontotemporal Dementia (FTD).

### MBI and functional brain imaging

Findings from fMRI studies [38, 40, 49, 55] have revealed MBI-specific neural network abnormalities associated with AD and FTD, and higher MBI burden was linked to reduced segregation of the salience [38] and frontoparietal control networks [40]. Despite being based on a cross-sectional perspective, these findings further support the potential involvement of AD or FTD processes in MBI onset.

While no significant associations were observed between MBI diagnosis and p-tau181 levels, hippocampal volumes, or entorhinal cortex thickness [35, 53, 54, 56], meta-analyses involving 1,225 and 752 participants, respectively, demonstrated cross-sectional positive correlations between MBI burden and brain deposition of Aß and tau [25, 26, 46]. Beyond the limited statistical power of the analyses due to the small number of studies included in each meta-analysis, our results may suggest that the severity of neuropsychiatric symptoms, as assessed through MBI, might be used as a clinical marker of the very early phase of AD, during which only Aβ- and tau-PET positivity is present, without yet showing biological signs of neurodegeneration [58]. In accordance with this hypothesis, Sun et al. observed a faster Aβ brain accumulation over 6 years among individuals with MBI, while no significant associations were found in relation to the speed of tau brain accumulation and decreased cerebral metabolism [46]. These apparently contrasting results can be explained by the amyloid cascade hypothesis, which posits that amyloid brain accumulation precedes – and potentially accelerates – tau pathology, ultimately leading to synaptic dysfunction and neurodegeneration [59]. 

### Strengths

This review is strengthened by its extensive search, conducted using a systematic methodology that follows PRISMA guidelines. Moreover, despite the limited number of studies included in the quantitative synthesis, this work presents the first meta-analysis on MBI and its neurobiological correlates – a novel and still underexplored topic.

### Limitations

Several limitations should be acknowledged when interpreting the findings of this review.

First, although the matching outcomes were quantitively evaluated, substantial heterogeneity in the assessment of neuropsychiatric symptoms (timing of onset, assessment tool and cut-off thresholds), reduces the reliability of pooled estimates and may inflate between-study variance. On this regard, although MBI diagnoses were made according to ISTAART criteria, studies adopted either prospective or retrospective approaches. Retrospective diagnoses could lead to overestimating events by including chronic symptoms or, conversely, underestimating them if the scale used assessed a shorter period (one month by NPI and NPI-Q), potentially excluding intermittent but persistent symptoms, with a possible misclassification bias. However, this bias may have influenced the results underestimating the differences in neurobiological findings between individuals with and without MBI. Additionally, we found significant variability in the evaluation of neuropsychiatric symptoms across studies, making direct comparisons difficult. Most research utilized the brief form of NPI (NPI-Q) and derived MBI from ten of the twelve items based on the conversion matrix by Morty et al. [16] Nevertheless, NPI was primarily designed to measure neuropsychiatric symptoms among individuals with dementia [27] and may not detect subtle neuropsychiatric symptoms in cognitively unimpaired adults or those with MCI, with a consequent underestimation of MBI prevalence. The MBI-C rating scale, used in 44.4% of the selected studies, was explicitly developed for screening MBI and is preferred for standardized evaluation of neuropsychiatric symptoms in a pre-dementia population [22]. In addition to significant structural differences between the two tools, the requirements to detect MBI varied across studies. For studies using the NPI/NPI-Q, the presence of at least one domain was considered suggestive for MBI, in accordance with ISTAART criteria. Instead, studies using the MBI-C applied different cut-offs ranging from 5 to 8 depending on the population, highlighting the ongoing debate regarding the optimal threshold. The lack of standardized measures limited the robustness of the quantitative synthesis from harmonized data. Finally, among the selected studies, the clinical diagnosis of MBI was not only investigated as a dichotomous variable; seven studies examined neuropsychiatric symptoms as a continuous variable through MBI burden, thereby including individuals without MBI in the analyses. Moreover, we found high heterogeneity in the study samples, which included widely varying proportions of individuals with MCI (ranging from 0.0% to 100.0%). In this context, it would be interesting to assess possible effect modification of cognitive performance in the associations between MBI and neurobiological correlates, which tend to be more pronounced in individuals with MCI than in cognitively unimpaired adults. Unfortunately, most of the studies did not provide subgroup analyses by MCI, so that this issue could not be verified in the present review. Furthermore, some studies relied solely on Mini-Mental State Examination total score within the normal range to define cognitively unimpaired individuals, which carries the risk of including those with subthreshold cognitive symptoms yet al.ready with MCI in this group [68], overestimating the analyzed associations. Finally, concerning the outcomes of interest, the brain imaging measures were highly variable and often could not be harmonized, making it challenging to summarize the results. This issue was not observed with CFS and plasma biomarkers; however, the small number of available studies restricted the possibility of performing an overall quantitative synthesis. Taken together, these sources of heterogeneity weaken the strength and generalizability of the conclusions, emphasizing the need for standardized diagnostic criteria, uniform cut-off thresholds, and harmonized outcome measures in future research.

Second, the limited number longitudinal studies, each exploring different outcomes, prevents direct comparisons. Only longitudinal evidence on MBI and changes in neurobiological findings over time will provide definitive confirmation of its role as an early stage of disease.

Third, given its high prevalence, the neurobiological correlates of AD were the most investigated in the selected studies. Nevertheless, emerging evidence on MBI and other types of dementia (e.g., FTD, VaD, Lewy Body Dementia, and others) is available, highlighting the need for further research to explore their specific neurobiological underpinnings.

### Future perspectives and research priorities

A key gap in the current literature is the limited number of studies with longitudinal design, as well as their short follow-up periods when present. Thus, this systematic review can only support the cross-sectional association between MBI and specific neurobiological correlates of AD. Without longitudinal data, no evidence can be summarized to disentangle whether MBI is an effective early sign of dementia or rather a risk factor for the disease. To answer this question, the relationship between baseline MBI and changes in brain volumes, neural activity, and CFS or blood biomarkers levels over time should be further investigated in cognitively unimpaired populations. Additionally, the follow-up duration should be precisely planned to be long enough to capture the conversion to MCI or dementia within the AD continuum. MCI and MBI may represent early dementia manifestations along cognitive and behavioral axes, respectively, having common neurodegenerative features (e.g. abnormal fluid AD biomarkers and Aβ- and tau-PET deposition). While both show mesial-temporal atrophy typical of AD, MBI preferentially affects the frontal lobes, suggestive of atypical AD and FTD. Unlike MCI, so far MBI has not been linked to cerebral vascular changes, such as white matter alterations. Moreover, regarding comorbid MCI, while it is well-established that the presence of MBI accelerates cognitive decline in MCI, few studies have examined whether it leads to a more rapid increase in AD biomarkers or more severe atrophy in this specific population. Ismail et al. conducted the only available longitudinal study on this issue: MBI was associated with a steeper 4-year increase in CFS p-tau levels both in ADNI and MEMENTO cohorts [27]. Additionally, in ADNI, MBI was also associated with a lower Aβ-42/40 ratio and a greater increase in t-tau levels over time [27]. These promising findings should be confirmed by future longitudinal studies with longer follow-ups, incorporating the evaluation of p-tau217, which has not yet been investigated.

**Fig. 3:**
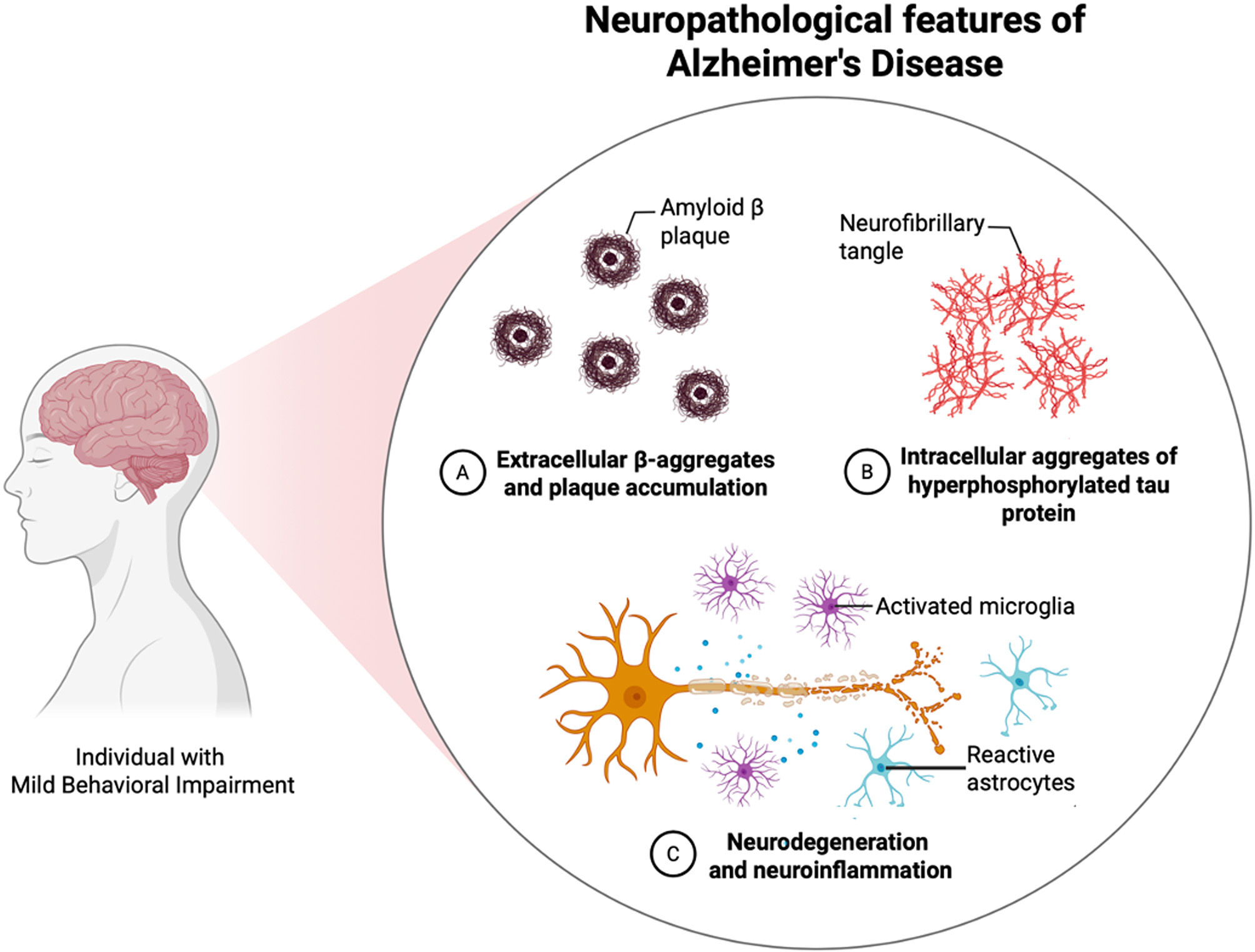
Pathophysiological hypothesis of Mild Behavioral Impairment. Created in BioRender. Remelli, F. (2025) https://BioRender.com/dcywics

Despite the consistent methodological heterogeneity across studies, our results support the role of MBI as an early sign of dementia, particularly AD, like MCI, but on the behavioral axis (Fig. 3). In addition to the neuropsychological batteries, the standardized neuropsychiatric evaluation – using MBI-C – might be performed for screening MBI in all individuals with initial cognitive-behavioral complaints. This approach may offer a simple and cost-effective way to identify individuals likely in the early stages of disease and those at high risk of progression from MCI to dementia [16, 19, 24, 69], thereby supporting physicians to identify who requires more advanced diagnostics and more frequent follow-up schedule [7, 16]. 

## Conclusions

The presence of MBI seems to be linked to specific neurobiological correlates of AD, including Aβ and tau brain deposition, as well as mesolimbic pathway injuries and abnormal neurodegenerative and neuroinflammatory fluid biomarker levels. Although growing evidence suggests that MBI might be considered a novel clinical marker of early AD, the considerable heterogeneity among studies does not permit solid and definitive conclusions. Further longitudinal studies with larger cohorts using harmonized methodologies are needed to clarify the biological correlates of MBI to explain its actual role in AD onset and speed of disease progression.

## Supplementary Information

Below is the link to the electronic supplementary material.

Supplementary Material 1

## Data Availability

All data generated during this systematic review are included in this published article.

## Abbreviations

AD: Alzheimer’s Disease
ISTAART: Alzheimer’s Association International Society to Advance Alzheimer’s Research and Treatment
Aβ42/40: Amyloid-beta 42/40 ratio
Aβ-PET: Amyloid Positron Emission Tomography
CFS: Cerebrospinal Fluid
DAT-SPECT: Dopamine Transporter Single Photon Emission Computed Tomography
DTI: Diffusor Tension Imaging
FDG-PET: 18 F-fluorodeoxyglucose Positron Emission Tomography
FTD: Frontotemporal Dementia
fMRI: Functional MRI
GFAP: Glial fibrillary acidic protein
I2: I-squared statistic
iRBD: Isolated REM sleep behavior disorder
MBI: Mild Behavioral Impairment
MBI-C: MBI-Checklist
MCI: Mild Cognitive Impairment
MRI: Magnetic Resonance Imaging
NfL: Neurofilament light chain
NPI: Neuropsychiatric Inventory
NOS: Newcastle-Ottawa Scale
NIA-AA: National Institute on Aging and Alzheimer’s Association
p-tau181: Phosphorylated-tau181
PD: Parkinson’s Disease
PRISMA: Preferred Reporting Items for Systematic Reviews and Meta-Analyses
tau-PET: Tau Positron Emission Tomography
t-tau: Total-tau
VaD: Vascular Dementia
$$\:\beta\::$$: Beta coefficients
95%CI: 95% Confidence Interval
